# The IL-17B-IL-17 receptor B pathway promotes resistance to paclitaxel in breast tumors through activation of the ERK1/2 pathway

**DOI:** 10.18632/oncotarget.23008

**Published:** 2017-12-06

**Authors:** Emilie Laprevotte, Stéphanie Cochaud, Stanislas du Manoir, Marion Lapierre, Cécile Dejou, Marion Philippe, Jérome Giustiniani, Kathryn A. Frewer, Andrew J. Sanders, Wen G. Jiang, Henri-Alexandre Michaud, Pierre-Emmanuel Colombo, Armand Bensussan, Gilles Alberici, Jérémy Bastid, Jean-François Eliaou, Nathalie Bonnefoy

**Affiliations:** ^1^ OREGA Biotech, Ecully, F-69130 France; ^2^ IRCM, INSERM, Université de Montpellier, ICM, Montpellier, F-34298 France; ^3^ Institut National de la Santé et de la Recherche Médicale (INSERM) UMR-S 976, Université Paris Diderot, Sorbonne Paris Cité, Laboratoire Immunologie Dermatologie and Oncologie, Paris, F-75475 France; ^4^ Institut Jean Godinot, Unicancer, Reims, F-51726 France; ^5^ Cardiff China Medical Research Collaborative, Cardiff University School of Medicine, Cardiff, CF14 4XN, UK; ^6^ Département de chirurgie oncologique, Institut Régional du Cancer de Montpellier, Université de Montpellier, Montpellier, F-34298 France; ^7^ Département d’Immunologie, Centre Hospitalier Régional Universitaire de Montpellier et Faculté de Médecine, Université de Montpellier, F-34295 France

**Keywords:** interleukin-17, interleukin-17B, breast cancer, chemoresistance, paclitaxel

## Abstract

Interleukin 17B (IL-17B) is a pro-inflammatory cytokine that belongs to the IL-17 cytokines family and binds to IL-17 receptor B (IL-17RB). Here we found that high expression of IL-17B and IL-17RB is associated with poor prognosis in patients with breast cancer and that IL-17B expression upregulation is specifically associated with poorer survival in patients with basal-like breast cancer. We thus focused on IL-17B role in breast cancer by using luminal and triple negative (TN)/basal-like tumor cell lines. We found that IL-17B induces resistance to conventional chemotherapeutic agents. *In vivo*, IL-17B induced resistance to paclitaxel and treatment with an anti-IL-17RB neutralizing antibody completely restored breast tumor chemosensitivity, leading to tumor shrinkage. We next focused on the signaling pathways activated in human breast cancer cell lines upon incubation with IL-17B. We observed that IL-17B induces ERK1/2 pathway activation, leading to upregulation of anti-apoptotic proteins of the BCL-2 family. IL-17B-induced chemoresistance was completely abolished by incubation with PD98059, an inhibitor of the MAPK/ERK pathway, indicating that the ERK pathway plays a crucial role. Altogether our results emphasize the role of the IL-17B/IL-17RB signaling pathway in breast tumors and identify IL-17B and its receptor as attractive therapeutic targets for potentiating breast cancer chemotherapy.

## INTRODUCTION

Tumor development is regulated by many different factors that influence cancer cell proliferation, cell death resistance and dissemination within the organism [1]. Particularly, it is now well established that inflammation-associated factors, such as chemokines and cytokines present within the tumor microenvironment, are crucial for modulating cancer progression and resistance to treatments [2]. Among the inflammatory mediators, a growing body of evidence emphasizes the contribution of the IL-17 cytokine family in malignant diseases. The IL-17 family includes six members (IL-17A to IL-17F) with different homology and functions [3]. IL-17A and IL-17F are the closest members, with 50% homology. These cytokines exert their activities through binding to IL-17 receptor (IL-17R) family members (IL-17RA to IL-17RE) that function as homo-or heterodimer complexes [4]. IL-17A is the prototypic member of the IL-17 family. It is predominantly produced by T helper 17 cells, a subset of CD4+ T cells. Binding of IL-17A to its receptor IL-17RA/RC leads to the production of cytokines and chemokines, such as TNF-α, IL-6, CXCL8 and CXCL1, that are involved in mechanisms of host defenses against extracellular bacterial and fungal infections [5]. Over-production of IL-17A has also been associated with chronic inflammatory disorders and autoimmune diseases [4, 6, 7]. Moreover, IL-17A has been detected in several human tumors, where it has been associated with pro-tumorigenic effects, and particularly with resistance to cancer therapies, by acting on cancer cells and also on the tumor microenvironment [8, 9].

Other members of the IL-17 and IL-17R families are expressed in various tissues at low levels. Although their involvement in pathological processes remains to be fully characterized, several experimental findings strongly suggest a role for the IL-17B/IL-17RB pathway in tumorigenesis. Indeed, the *IL-17RB* locus is a common site of retroviral integration in murine myeloid leukemia. The resulting IL-17RB expression upregulation in leukemic cells suggests a pro-oncogenic role for this receptor [10]. In humans, IL-17RB overexpression has been reported in a subset of adult patients with acute T-cell leukemia. Moreover, IL-17RB overexpression in HTLV-1 transformed T cells contributes to cell growth and survival *via* the NF-κB pathway [11]. In solid tumors, amplified IL-17B/IL-17RB signaling is critical for breast and pancreatic tumorigenesis and elevated IL-17RB expression has been associated with the shortest survival rates in patients with breast or pancreatic cancer. Specifically, IL-17B produced by breast cancer cells promotes anti-apoptotic signaling and tumor survival through activation of the NF-κB pathway [12]. In pancreatic cancer cell lines, IL-17B activates the ERK1/2 pathway and induces the production of the chemokines CCL20, CXCL1, IL-8 and TFF1. Following the activation of such chemokines, IL-17B promotes the recruitment of macrophages that, in turn, favors cancer cell survival and invasion as well as the recruitment of endothelial cells with vasculogenic potential, thus stimulating tumor angiogenesis [13].

Thus, besides IL-17A, IL-17B also could play an important role in tumor progression through its binding to IL-17RB and might represent a potential therapeutic target in solid tumors, such as breast and pancreatic cancers. As IL-17B role in the response to anti-cancer therapies has not been precisely investigated yet, we decided to explore IL-17B function in breast tumor response to chemotherapy.

## RESULTS

### IL-17B overexpression in breast cancer is associated with poor prognosis

**Figure 1:**
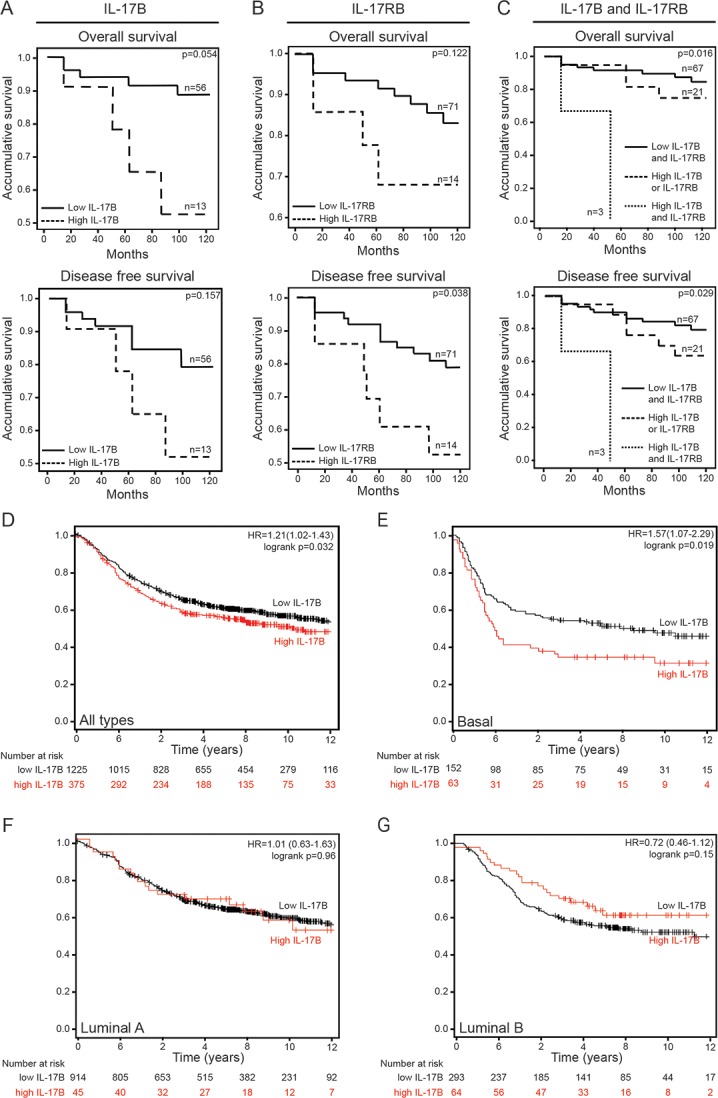
IL-17B/IL-17RB overexpression correlates with poor clinical outcome in patients with breast cancer (**A–C**) Comparison of the overall survival and disease-free survival rate in a cohort of patients (*n* = 143) with breast cancer that express different levels of IL-17B (A), IL-17RB (B) or both IL-17B and IL-17RB (C) using the Kaplan-Meier method and the Wilcoxon test. (**D–G)** Prognostic effect of IL-17B expression in the whole population (D) and in patients with basal-like (E), luminal A (F) or luminal B (G) breast cancer assessed using the Kaplan-Meier method. Survival data were compared using the log-rank test. The third quartile was taken as the threshold for high IL-17B expression. Cohort size = 1809 patients, database 2012, collection of Affymetrix chips.

Although high IL-17RB expression was previously associated with poor prognosis in patients with breast cancer [12, 14], the prognostic value of IL-17B *per se* has never been investigated. We thus evaluated the expression of IL-17B and IL-17RB by real-time quantitative PCR in biopsies from a cohort of patients with breast cancer (*n* = 143) (Supplementary Table 1) and 10 years of follow-up. We then assessed the correlation between IL-17B and IL-17RB expression and patient survival. High and low IL-17B or IL-17RB expression levels were defined based on a cutoff value calculated from the internal standard curve. As indicated by the Kaplan-Meier survival analysis in Figure 1A and 1B, high IL-17B expression tended to be associated with reduced overall survival (OS) (*p* = 0.05), but not disease-free survival (DFS) (*p* = 0.15). Conversely, high IL-17RB levels were associated with reduced DFS (*p* = 0.03). Moreover, OS (*p* = 0.016) and DFS (*p* = 0.029) were significantly reduced in patients with cancers in which both IL-17B and IL-17RB were overexpressed compared with patients with cancers where only one was upregulated or where both molecules were expressed at low level (Figure 1C). Although these data must be confirmed in a larger cohort of patients, they indicate that in addition to IL-17RB, IL-17B expression level also could influence breast cancer prognosis.

To further refine the prognostic value of IL-17B *per se,* we next analyzed the microarray results of a cohort of 1809 patients with breast cancer [15]. Kaplan-Meier graphs for recurrence-free survival in the whole population or only in patients with luminal A, luminal B or basal-like breast cancer showed that high IL-17B expression was significantly correlated with poorer prognosis in the whole population (Figure 1D, *p* = 0.032 and 15% probability decrease) and in the basal-like subtype (Figure 1E, *p* = 0.019 and 15% probability decrease), but not in the other subtypes (Figure 1F and 1G). In contrast, high expression of IL-17A and IL-17E was associated with favorable outcomes in the whole population as well as luminal A, luminal B or basal-like molecular subtypes (Supplementary Figure 1).

**Figure 2:**
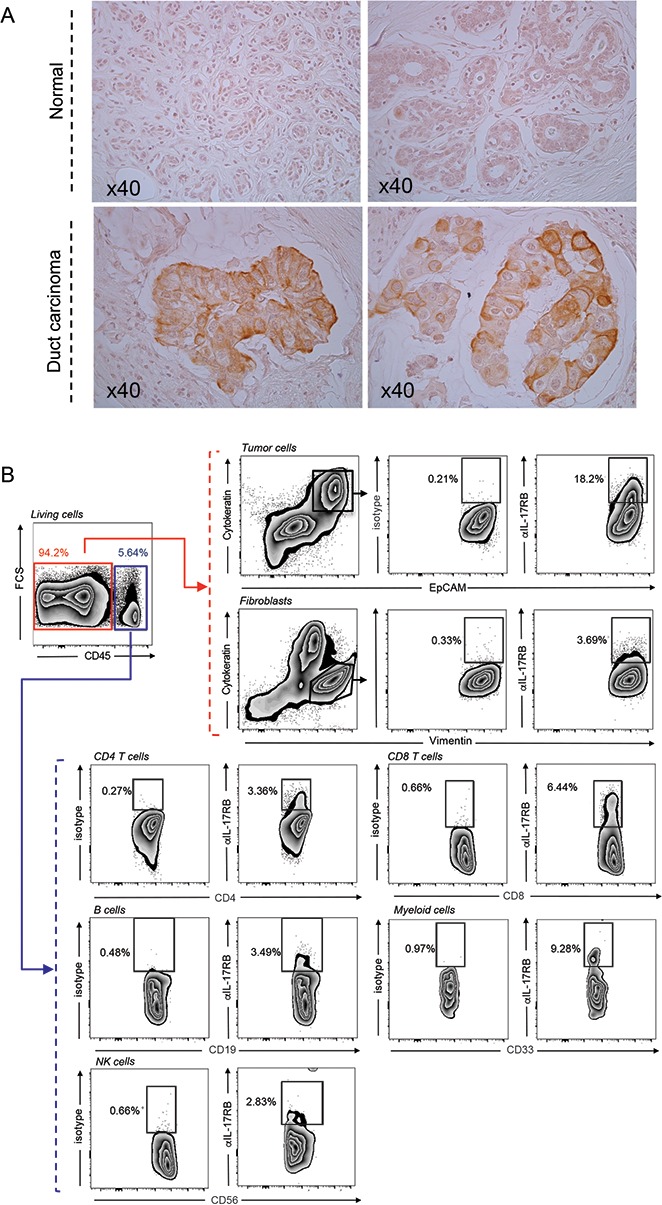
Expression of IL-17B and IL-17RB in breast tumors **(A)** Representative immunohistochemical staining of IL-17B expression in normal and breast cancer tissues. (**B)** Representative gating strategy and flow cytometry contour plots showing the percentages of IL-17RB expressing cells in a fresh dissociated breast tumor biopsy from a patient with invasive carcinoma. Percentages of IL17-RB^+^ cells were determined on CD45^–^Cytokeratin^+^EpCam^+^ tumor cells, CD45^–^Cytokeratin^-^Vimentin^+^ fibroblasts and within the CD45^+^ cells on CD4^+^ and CD8^+^ T cells, CD19^+^ B cells, CD33^+^ myeloid cells and CD56^+^ NK cells.

Importantly when we examined IL-17B expression on a human tissue array of breast cancer and metastasis, we found that IL-17B is mainly expressed by the tumor cells (Figure 2A). Reinforcing such observation, qRT-PCR analysis of TILs expanded from 4 breast cancer patients’ biopsies showed that all the 4 samples were negative or limit to detection (TIL AL) for IL-17B mRNA expression, while half of them expressed IL-17A mRNA (Supplementary Figure 2).

On the contrary, analysis on a fresh breast tumor biopsy, by flow cytometry, revealed that IL-17RB was expressed by the EpCAM^+^ Cytokeratin^+^ tumor cells and by some fibroblasts, but also immune subpopulations within the tumor microenvironment (Figure 2B).

### IL-17B promotes resistance to paclitaxel *in vitro* via activation of the ERK pathway

**Figure 3:**
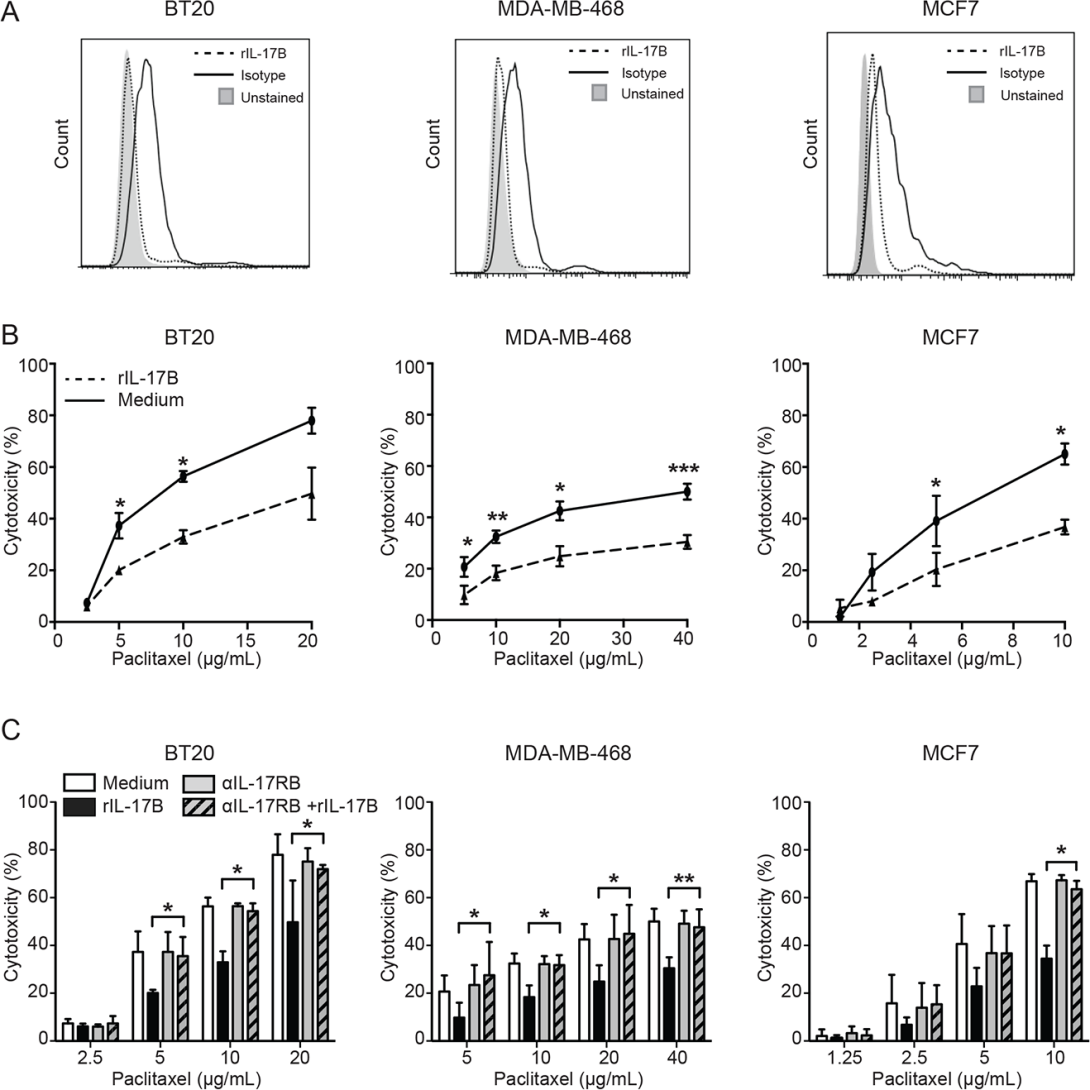
IL-17B promotes resistance to paclitaxel *in vitro* (**A**) Expression of IL-17RB BT20, MDA-MB-468 and cells MCF7 determined by flow cytometry. (**B)** Percentages of drug-induced cell cytotoxicity in BT20, MDA-MB-468 and cells MCF7 cultured in FCS-free medium completed or not with 10 ng/mL rIL-17B for 24 h and treated with paclitaxel at concentrations ranging from 2.5 µg/mL to 40 µg/mL for 7 h. (**C)** Same as in B, except that cells were cultured in FCS-free medium completed or not with rIL-17B and/or anti-IL-17RB antibody (10 µg/mL) before treatment with paclitaxel. The Student’s *t-test* was used to compare control and treatment groups; ^*^p < 0.05 ^**^p < 0.01 and ^***^p < 0.001.

We next investigated whether activation of the IL-17B/IL-17RB pathway was involved in the development of resistance to conventional chemotherapeutic agents, such as paclitaxel, in breast tumors. Thus, various human breast cancer cell lines, in which IL-17RB expression was confirmed by flow cytometry (Figure 3A), were cultured in the presence of 10 ng/mL of recombinant human IL-17B (rIL-17B) for 72 h and then incubated with paclitaxel at different concentrations. Pre-treatment with rIL-17B significantly decreased paclitaxel-induced cytotoxicity in BT20 and MDA-MB-468 cells (derived from TN/basal-like breast cancers) and also in MCF7 cells (from a luminal A breast cancer) (Figure 3B), while it had not effect on the IL-17RB negative MDA-MB-435S cell line (Supplementary Figure 3A and 3B). Resistance to paclitaxel was strictly dependent on IL-17B/IL-17RB interaction because addition of an anti-IL-17RB neutralizing antibody to the culture medium restored sensitivity to paclitaxel (Figure 3C).

**Figure 4:**
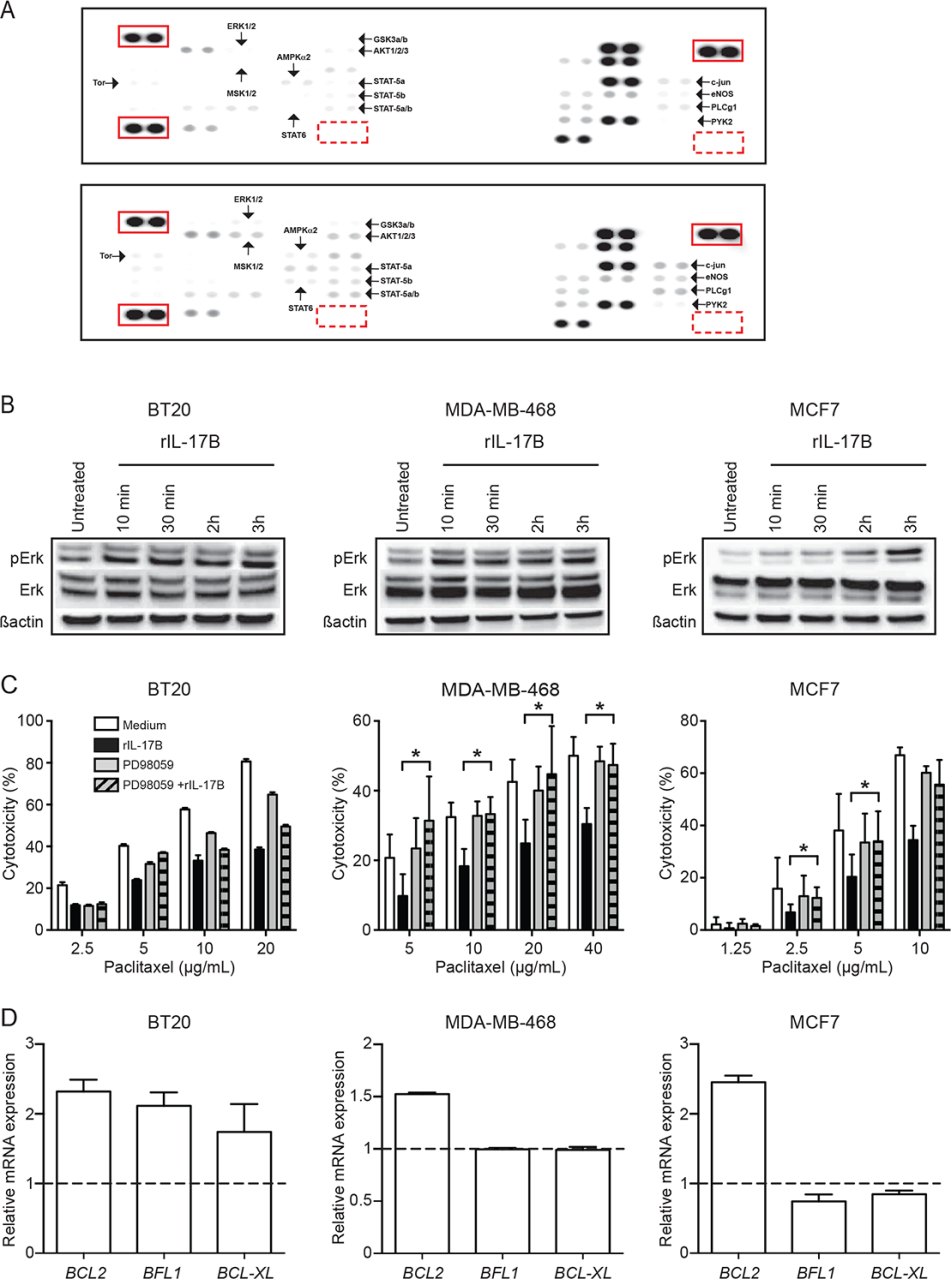
IL-17B-induced resistance to paclitaxel is mediated by activation of the ERK pathway **(A)** Human phospho-kinase array of BT20 cultured in the presence of medium alone or rIL-17B 10 ng/ml for 30 min. Red squares identify reference spot/positive (full lane) and PBS/negative (dotted lane) controls. Arrows indicate proteins that are differentially phosphorylated between the 2 conditions. (**B)** Western blot analysis of ERK1/2 phosphorylation (pErk) in BT20, MDA-MB-468 and cells MCF7 stimulated or not with 10 ng/mL rIL-17B for different times. Total ERK and β-actin were used as protein loading control. Results are representative of at least 3 experiments. (**C)** Percentages of drug-induced cell cytotoxicity in BT20, MDA-MB-468 and cells MCF7 cultured in FCS-free medium completed or not with 10 ng/mL rIL-17B and/or 25 µM PD98059 (MAPK/ERK1/2 inhibitor) for 24 h before treatment with paclitaxel and treated with paclitaxel at concentrations ranging from 2.5 µg/mL to 40 µg/mL for 7 h. The Student’s *t-*test was used to compare control and treatment groups; ^*^p < 0.05. (**D)** Expression of *BCL2*, *BFL1* and *BCL-XL* mRNA in BT20, MDA-MB-468 and MCF7 cells stimulated with 10 ng/mL rIL-17B for 24 h. Values represent mean ± SD of 3 independent experiments; dashed line represents the level of expression of the target gene in untreated cells.

To elucidate the mechanisms involved in IL-17B-induced resistance to paclitaxel, we first compared the kinase activation profile of BT20 cells cultured with or without rIL-17B using a phosphokinase antibody array. We identified several candidate proteins the activity of which was positively regulated by IL-17B, such as AKT, STAT5a/b, ERK1/2 and MSK-1/2 a downstream target of ERK1/2 (Figure 4A). Activation of the ERK1/2 pathway by rIL-17B was reminiscent to our previous observations with IL-17A, and we indeed confirmed that both cytokines induced similar ERK1/2 activity in BT20 cells as revealed by phosphorylation of ERK1/2 analyzed by western blotting using specific antibodies (Supplementary Figure 3C). Analysis of phosphorylation of ERK1/2 showed that, although the kinetics were not similar, ERK1/2 activity was consistently increased by rIL-17B in the three breast cancer cell lines BT20, MDA-MB-468 and MCF7, (Figure 4B), but not in the IL-17RB deficient MDA-MB-435S cell line (Supplementary Figure 3D). To further support the role of ERK1/2 activation in IL-17B-induced resistance to paclitaxel, we inhibited the ERK1/2 pathway in the three cell lines using PD98059, a chemical inhibitor of the MAPK/ERK cascade. PD98059 abrogated rIL-17B-induced resistance to paclitaxel in MDA-MB-468 and MCF7 cells (Figure 4C). Conversely, resistance to paclitaxel was only partially reverted in BT20 cells upon incubation with PD98059, suggesting the implication of a more complex signaling network in this cell line. For instance, we observed that rIL-17B also activated the NF-κB pathway in BT20 (Supplementary Figure 3E), but not in MCF7 and MDA-MB-468 cells. However, the pharmacological NF-κB inhibitors CAPE and BAY11-7082, used at non-toxic concentrations, did not abrogate rIL-17B effect in BT20 cells.

Real-time quantitative PCR analysis revealed that incubation with rIL-17B upregulated the expression of anti-apoptotic genes: *BCL-2* in MDA-MB-468, BT20 and MCF7 cells, but also the two NF-κB-regulated genes *BFL-1* and *BCL-XL* [16, 17] in BT20 cells (Figure 4D).

These observations suggest that IL-17B might exert a pro-tumoral activity in breast cancer by modulating paclitaxel efficacy through the activation of the ERK1/2 pathway and upregulation of BCL-2 family members.

### IL-17B promotes resistance to paclitaxel *in vivo*

**Figure 5:**
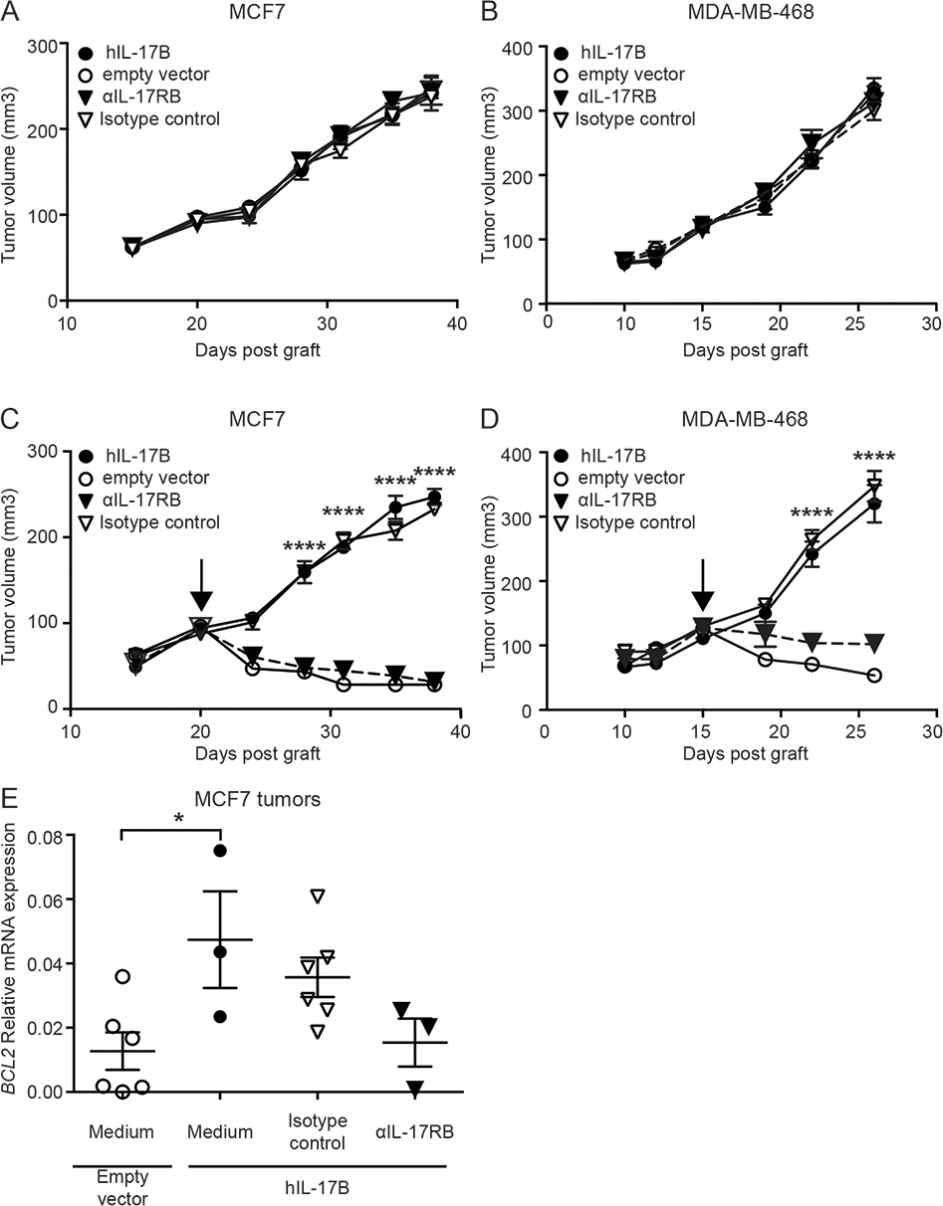
IL-17B promotes resistance to paclitaxel *in vivo* (**A, B**) Tumor growth of MCF7 (**A**) and MDA-MB-468 (**B**) cells expressing hIL-17B (hIL-17B) or not (empty vector) treated with anti-IL-17RB antibody (αIL-17RB) or its isotype control (IgG2b) (50 µg intratumor twice a week). Antibodies treatments were initiated when tumors reached about 100 mm^3^ in size. (**C, D)** Same as in A and B except that mice received also paclitaxel (10 mg/kg twice a week). Each group consisted of 10 mice, results are representative of at least 2 experiments. (**E)** *BCL2* mRNA expression in MCF7 tumors at sacrifice (values for each individual mouse are indicated in the graph). The 2-way ANOVA test and the corrected original False Discovery Rate (original method of Benjamini and Hochberg) multiple comparisons test were used to compare tumor growth in mice engrafted with MCF7 and MDA-MB-468 cells expressing hIL-17B treated with isotype control *vs* αIL-17RB and the Student’s *t-*test was used to compare mRNA expression; ^*^p < 0.05 and ^****^p < 0.0001.

To evaluate IL-17B effect on resistance to paclitaxel *in vivo*, we xenografted nude mice with MCF7 or MDA-MB-468 cells that express or not hIL-17B. In agreement with the absence of rIL-17B effect on MCF-7 and MDA-MB-468 cell proliferation *in vitro* (Supplementary Figure 4A), constitutive IL-17B expression in MCF7-h-IL17B and MDA-MB-468-h-IL-17B cells did not have any effect on their expansion *in vitro* (Supplementary Figure 4B) and their tumor growth *in vivo*. Tumor size and growth kinetics were similar in nude mice engrafted with hIL-17B expressing cells or control cells (Figure 5A and 5B). Paclitaxel treatment (10 mg/kg twice a week), when tumors reached a volume of about 100 mm^3^, induced tumor regression only in mice grafted with control cells, whereas the growth of MCF7-hIL-17B and MDA-MB-468-hIL-17B tumors was not affected (Figure 5C and 5D). Moreover, administration of a neutralizing anti-IL-17RB antibody (50 µg/injection twice a week) did not affect MCF7-hIL-17B and MDA-MB-468-hIL-17B tumor progression in the absence of paclitaxel treatment (Figure 5A and 5B). Conversely, it restored their sensitivity to paclitaxel, while the control isotype antibody had no effect (Figures 5C and 5D). Although there is no validated biomarker to predict resistance to taxanes in breast cancer, acquired resistance to paclitaxel in MCF-7 cells has been associated with BCL-2 upregulation. [18] In agreement, we observed that *BCL-2* expression was significantly higher in tumors resistant to paclitaxel (mice xenografted with MCF7-h-IL17B cells) compared with sensitive tumors (mice xenografted with MCF-7 control cells, empty vector). Moreover, restoration of paclitaxel sensitivity in MCF7-h-IL17B cells following the treatment with the anti-IL-17RB neutralizing antibody was associated with *BCL-2* expression downregulation (Figure 5E).

## DISCUSSION

We demonstrated here that IL-17B, through binding to IL-17RB, promotes paclitaxel resistance in luminal and TN/basal-like breast cancer cells *in vitro* and *in vivo* and brought the first proof of concept that neutralization of the IL-17B/IL-17RB pathway could sensitize breast tumors to paclitaxel therapy. Together with the demonstration that IL-17RB signaling is associated with *in vitro* resistance to etoposide in breast cancer cell lines [12], our results indicate that the IL-17B/IL-17RB pathway can modulate the cytotoxic effect of drugs that act through at least two independent mechanisms: topoisomerase II inhibition (etoposide) and spindle poisoning (paclitaxel). As we previously reported for IL-17A [8], IL-17B present in the tumor microenvironment could be an important cancer cell survival factor and a mediator of therapy resistance in breast cancer. Interestingly, in contrast to IL-17A, which is produced by TILs, the main source of IL-17B in breast tumors seems to be tumor cells themselves and potentially fibroblasts, but not TILs (Figure 2 and Supplementary Figure 2).

Huang *et al.* previously reported that disruption of the IL-17B/IL-17RB pathway in breast cancer cell lines negatively affects colony formation in soft agar assays. However, this effect was cell line-dependent, because IL-17B did not promote colony formation in cell lines that express low IL-17RB levels, such as MDA-MB-231 cells. Although we did not perform colony formation assays, we observed that rIL-17B has a cell line-dependent effect on breast cancer cell proliferation (Supplementary Figure 4A). Moreover, the absence of rIL-17B effect on MCF7 and MDA-MB-468 proliferation indicates that IL-17B promotion of chemotherapy resistance can be clearly dissociated from its effect on cell proliferation. These results are strengthened by the observation that cell proliferation was comparable in MCF7 and MDA-MB-468 cells transfected with the hIL-17B-pcDNA3 vector to express human IL-17B (MCF7-hIL-17B and MDA-MB-468-hIL-17B cells) and in MCF-7 and MDA-MB-468 cells transfected with pcDNA3 vector alone (Supplementary Figure 4B). Conversely, MCF7-hIL-17B and MDA-MB-468-hIL-17B cells showed a high level of resistance to paclitaxel *in vitro*, comparable with the effect observed following incubation with rIL-17B in parental MCF7 and MDA-MB-468 cells (Supplementary Figure 4C). Altogether these results emphasize the specific effect of IL-17B on resistance of breast cancer cell lines to paclitaxel-based chemotherapy.

Our results could be important particularly for patients with TN breast cancer who cannot benefit from the current targeted therapies and whose initial clinical response to neoadjuvant chemotherapy determines their prognosis. The clinical relevance of IL-17B overexpression in the basal-like subtype is supported by microarray analyses showing poor relapse-free survival in patients with high IL-17B mRNA level. The observation that IL-17B effects were not limited to basal-like breast cancer cell lines suggests that the IL-17B/IL-17RB role in TN/basal-like breast tumors is mainly due to upregulation of this pathway rather than to TN/basal-like-specific mechanisms. However, the molecular mechanisms governing IL-17B expression upregulation in breast cancer cells or in their microenvironment are unknown and they deserve additional investigations.

The basal-like subtype includes most (70–80%) TN breast tumors [19] that are not eligible for hormonotherapy and treatment with herceptin/trastuzumab. Although TN breast cancers are generally associated with poor outcome, they are not thought to be resistant to chemotherapy and anthracycline/taxane-based neoadjuvant chemotherapy is considered the standard-of-care regimen. With this treatment, pathological complete remission is obtained in about 22% of patients. However, early relapses are common and patients with TN breast cancer and residual disease are considered at high risk of recurrence and metastases [20, 21]. These clinical observations emphasize the importance of improving the initial response to chemotherapy by identifying and neutralizing resistance mechanisms.

Finally, our results emphasize the role of the IL-17B/IL-17RB signaling pathway in breast cancer and identify IL-17B and its receptor as attractive therapeutic targets for therapy. Nevertheless, the specific role of IL-17B compared with other IL-17 family members, especially IL-17A, needs to be precisely investigated to develop adapted therapeutic strategies.

## MATERIALS AND METHODS

### Breast cancer patients’ analysis

In the first cohort, overall survival (OS) and disease-free survival (DFS) rates of breast cancer patients (*n* = 143) that express different levels of IL-17B, IL-17RB or both IL-17B and IL-17RB were analyzed using the Kaplan-Meier method and the Wilcoxon test. Ethical approval was obtained from the Brotaf Health Authority (References 01/4046 and 01/4303) and all patients signed a written informed consent. In the second cohort, the prognostic effect of IL-17B expression in the whole population and in patients with luminal A, luminal B or with basal-like breast cancer was assessed using the Kaplan-Meier method. Survival data were compared using the log-rank test. The threshold of expression value giving the best RFS separation between high and low expression groups was first calculated, then the closest value among the median, tertiles and quartiles was retained as current threshold for analysis including each molecular subtype. The third quartile was taken as the threshold for high IL-17B expression. Cohort size = 1809 patients, database 2012, collection of Affymetrix chips (threshold 20). The median was taken as the threshold for high IL-17A expression. Cohort size = 1809 patients, database 2012, collection of Affymetrix chips (threshold 50). The median was taken as the threshold for high IL-17E expression. Cohort size = 1809 patients, database 2012, collection of Affymetrix chips (threshold 35).

### Cell culture and reagents

BT20, MCF7and MDA-MB-cell lines were obtained from ATCC. For all experiments, cells were cultured at 37°C and 5% CO2. BT20 and MCF7 were cultured in RPMI supplemented with 10% heat-inactivated fetal calf serum (FCS), 2 mM L-glutamine and 40 µg/mL streptomycin. MDA-MB-468 were cultured in DMEM supplemented with 10% heat-inactivated FCS, 2 mM L-glutamine and 40 µg/mL streptomycin. All media and reagents were purchased from Invitrogen. Recombinant Human IL-17B (rIL-17B) (Peprotech) was used at a final concentration of 10 ng/mL, anti-IL-17B receptor antibody (RD systems) at 10 µg/mL, MAPK/Erk-inhibitor PD98059 (InvivoGen, San Diego, USA) at 25 µM. Paclitaxel was obtained from the Institut du Cancer de Montpellier and was used at a range of concentrations varying from 2.5 µg/mL to 40 µg/mL depending on cell lines.

### Clinical breast cancer biopsies

Human biopsy samples were obtained from the cancer center Institut Jean Godinot or from the Institut regional du Cancer de Montpellier. All patients were informed and agreed the study. Tumor was cut in small pieces of 0.5 cm^3^ in RPMI 1640 medium and dissociated by combining mechanical disruption (GentleMACSTM dissociator, Miltenyi Biotec) with enzymatic digestion using 500 µg/ml collagenase type IV- S (Sigma). After dissociation, sample was filtered (40 µm nylon filter) to remove any remaining larger particles from the cell suspension and the recovered cells were used immediately either for isolation and expansion of tumor-infiltrating lymphocyte (TIL) and cancer-associated fibroblasts as previously described [8] or for flow cytometry analysis.

### Flow cytometry analysis

For flow cytometry analysis, cells were washed once with PBS and antibodies for surface markers were added at 4°C for 1 hour. When required, after washes cells were fixed and permeabilized, according to the BD Biosciences fixation and permeabilization procedures, and intracellular staining (vimentin and cytokeratin) was performed overnight at 4°C. Then, samples were washed, fixed in 1% PFA, and processed for data acquisition using a Cytoflex flow cytometer (Beckton Dickinson). Data were analyzed using the Flowjo 10 software. The following antibodies were used: anti-human CD45-BV786 (clone HI30, BD Horizon), anti-human CD19-FITC (clone HB19, Biolegend), anti-human CD56-PE (clone N901, Beckman Coulter), anti-human CD33-AF700 (clone WM53, BD Pharmingen), anti-human CD8-BV605 (clone SK1, BD Horizon), anti-CD4-BV650 (clone SK3, BD Horizon), anti-human EPCAM-PE-CF594 (clone EBA-1, BD Horizon), anti-human IL-17RB-APC (clone #170220, R&D Systems).

### IL-17B immunohistochemistry

Tissue microarray (CBA4, Super Bio Chips, Clinisciences, Nanterre, France) was used to analyze the expression of IL-17B in breast tumors and in metastasis. The TMA sections were deparaffinized in xylene for 10 min, rehydrated in an ethanol gradient and heated at 99°C for 30 min in citrate buffer (pH 9.0) for antigen retrieval. Endogenous peroxidase was inactivated by incubating the sections in 0.3% H2O2 for 20 min at room temperature. The sections were blocked in PBS-goat serum 20% for 1 h at 37°C and incubated 1 h at room temperature with anti-IL-17B antibody (dilution, 1:50; LSB Bio, Seattle, US). Finally, the sections were stained with EnVision FLEX/HRP (Dako, Les Ulis, France) for 20 min at room temperature. Signal detection was carried out using a solution of DAB (vector SK4100; Vector laboratories, Burlingame, US). The TMA sections were counterstained with hematoxylin, dehydrated, and mounted.

### Generation of stable cell lines over-expressing human IL-17B (hIL-17B)

The human IL-17B cDNA was subcloned into pcDNA3/hygromycin vector. MCF7 and MDA-MB-468 cells were transfected with empty pcDNA3/hygromycin plasmid or with the hIL-17B-pcDNA3/hygromycin plasmid and selected with 225 µg/ml hygromycin B. The expression level of hIL-17B was confirmed by RT-qPCR.

### Isolation of mRNA and real-time quantitative PCR

Total RNA was isolated using the RNeasy Mini Kit (Qiagen, Courtaboeuf, France) and 1 μg of total RNA was reverse transcribed with SuperScript III (Invitrogen, Courtaboeuf, France) according to the manufacturer’s instructions. RT-qPCR analyses were performed using Sybr Premix Ex Tak (Tli RNase H plus, Takara Bio Europe/Clontech, Saint Germain en Laye, France) to determine the gene expression of *IL-17A, IL-17B*, *IL-17C, IL-17D, IL-17E,* IL-17F, *IL-17RA, IL-17RB, IL-17RC, IL-17RD, IL-17RE, BCL2*, *BFL-1*, *BCL-XL* and *GAPDH* with the following primers: IL-17A (Forward: 5′-ACTACAACCGATCCACCTCAC-3′; Reverse: 5′-ACTTTGCCTCCCAGATCACAG-3′), IL-17B (Forward: 5′-GCCACTGGACCTGGTGTCACG-3′; Reverse: 5′-CTGGGGTCGTGGTTGATGCTGT-3′), IL-17C (Forward: 5′-TGCCAAGTGGGGGCAGGCTT-3′; Reverse: 5′-CGTGTCCACACGGTATCTCCAGGG-3′), IL-17D (Forward: 5′-GCCCTGGGCCTACAGAATCTCCT-3′; Reverse: 5′-CCTCGGTGTAGACGGAACGGC-3′), IL-17E (Forward: 5′-TCCCCCTGGAGATATGAGTTGGACA-3′; Reverse: 5′-GGCATGGCCGCCGGTAGAAG-3′), IL-17F (Forward: 5′-TGGGCTCGATCAATGCTCAAGG-3′; Reverse: 5′-TCGACCTCTTACTGCACACGG-3′), IL-17RA (Forward: 5′-TGCCCCTGTGGGTGTACTGGT-3′; Reverse: 5′-GCAGGCAGGCCATCGGTGTA-3′), IL-17RB (Forward: 5′-TACCCCGAGAGCCGACCGTT-3′; Reverse: 5′-GGCATCTGCCCGGAGTACCCA-3′), IL-17RC (Forward: 5′-GGCTTGGTTTCACGCGCAGC-3′; Reverse: 5′-CGGCCCTGCAAGAAGTCGGG-3′), IL-17RD (Forward: 5′-ACACCTGTGGCTGGAGGGGAG-3′; Reverse: 5′-GCCACTTGGTCATGGCAAGCA-3′), IL-17RE (Forward: 5′-CCACCTTCAGGCCATGCAGCC-3′; Reverse: 5′-CTGTCATCCGTGTGGGAGGCCA-3′), BCL2 (Forward: 5′-TGTGGATGACTGAGTACCTGAACC -3′; Reverse: 5′-GTTTGGGGCAGGCATGTTGAC-3′), BFL-1 (Forward: 5′-ACAGGCTGGCTCAGGACTATCT-3′; Reverse: 5′- CTCTGGACGTTTTGCTTGGAC-3′), BCL-XL (Forward: 5′-CGTGGAAAGCGTAGACAAGGA-3′; Reverse: 5′-ATTCAGGTAAGTGGCCATCCAA-3′) and GAPDH (For^—^ward: 5′-GAAGGTGAAGGTCGGAGTCA-3′; Reverse: 5′-GACAAGCTTCCCGTTCTCAG-3′). Results were normalized to GAPDH housekeeping gene transcripts.

### Cytotoxicity assays

MCF7 (1,000 cells/well), BT20 and MDA-MB-468 (3,000 cells /wells) cells were seeded in a 96 wells plate in adequate complete medium alone or with rIL-17B for 48 h. Then medium was removed and replaced with FCS-free medium supplemented or not with IL-17B, and/or antibodies, and/or PD98059 when indicated for 24 h. Finally, cells were treated with paclitaxel at concentrations ranging from 2.5 µg/mL to 40 µg/mL, depending on the cell line, for 7 h. Untreated cells (control medium) and Triton X100 treated cells (100% cell death) were used as controls. Each condition was performed in triplicates. The cytotoxicity was determined using the Cytotoxicity Detection Kit (Roche) according to the manufacturer’s instructions. Percentage of cytotoxicity is calculated as followed: % = 100 ^*^ (exp value − control medium value)/(Triton X100 treated cells value − control medium value).

### Cell proliferation assays

MCF7, T47D, MDA-MB468m MDA-MB-436 (500 cells/well) and BT20 (1,000 cells/well) cells were plated on 96-well plates and maintained for 24 h in complete medium. Medium was then removed and replaced with complete medium supplemented with or without rIL-17B (Peprotech, Neuilly-sur-Seine, France). After 72 h of culture, cells were pulsed with 1 mCi of tritiated thymidine ([3H]-TdR) per well. [3H]-TdR uptake was measured using PerkinElmer PerkinElmer MicroBeta2plate counter (PerkinElmer, Waltham, MA, USA). Each experiment was performed in hexaplicates.

MDA-MB-468 and MCF7 cells overexpressing or not human IL-17B were plated in E-plate 16 (ACEA Biosciences, San Diego, USA) of the Xcelligence system at 3,000 cells/well in complete medium. Once cells were attached to the bottom of the E-plate 16, cell proliferation was monitored every 3 h and up to 150 h. Cell proliferation was expressed as a cell index recorded using the RTCA software 2.0. The system displays the real-time growth curves of cells.

### Phospho-kinase array

BT20 breast cancer cells were serum starved for 24 h and then stimulated with rIL-17B (10 ng/mL) for 30 min. Then whole protein extracts were prepared and analyzed using the Human Phospho-Kinase Array Kit (ARY003B, R&D System) according to manufacturer’s instructions.

### Immunoblotting

MCF7, BT20 and MDA-MB-468 breast cancer cells were serum-starved for 24 h and then stimulated with rIL-17B (10 ng/mL). Whole protein extracts were prepared using the M-PER Mammalian Protein Extraction Reagent kit (Thermo scientific, Courtaboeuf, France) and analyzed by western blotting using antibodies against phosphorylated ERK1/2 (p-ERK) (1/500), ERK1/2 (ERK) (1/1000) or β-actin (1/10,000) (Cell signaling, Ozyme, Saint Quentin en Yvelynes, France). Signals were revealed using horseradish peroxidase-conjugated secondary antibodies (Jackson ImmunoResearch, Interchim, Montlucon, France) and enhanced chemiluminescence (ECL-Plus, Perkin Elmer, Courtaboeuf, France) according to the manufacturer’s instructions.

### Xenografts in athymic nude mice

Eight-week-old female athymic nude *Foxn1*^nu^ mice (Nude mice) were purchased from Envigo. MCF7 or MDA-MB-468 cells (2 × 10^6^) with Matrigel (Matrigel matrix growth factors reduced, Corning, Bedford, USA) were injected subcutaneously into Nude mice. Tumor volumes were evaluated every 3 days after initial detection with a caliper. Intraperitoneal administration of taxol (10 mg/kg twice a week) or intra-tumoral injection of anti-IL-17RB antibody or its isotype control IgG2b (50 µg per tumor in 20 µL of sterile PBS twice a week) was initiated when tumors reached about 100 mm^3^ in size. All procedures for animal handling and experiments were approved by the local animal facility “ComEth” Institutional Review Board under the supervision of the French LR Regional CEEA ethics committee on animal experimentation (Chairman: Pr M Michel, Montpellier, France).

### Statistical analysis

Prism software version 7.0c (GraphPad Software, San Diego, CA) was used to analyze the data. For Figure 1, Kaplan-Meier survival curves were used to analyze the percentage survival of patients. For Figures 2,3,4E and Supplementary Figure 2C, cytotoxicity and relative mRNA expression were assessed with the two-tailed Student *t*-test. In Figure 4, tumor growth curve comparisons were done using a 2-way ANOVA test and the corrected original False Discovery Rate (original method of Benjamini and Hochberg) multiple comparisons test. All data were presented as mean ± SEM except when indicated specifically in figure legends. The effect of rIL-17B on cell proliferation (Supplementary Figure 2A) was assessed by the non-parametric Kruskal–Wallis test with Dunn’s post-test to compare treated *vs* untreated conditions. Statistical significance was indicated in figures with an asterisk where *p* < 0.05, two asterisks where *p* < 0.01 and three asterisks where *p* < 0.001 and four asterisks where *p* < 0.0001.

## SUPPLEMENTARY MATERIALS FIGURES AND TABLE



## Abbreviations

DFS: disease-free survival
IL: interleukin
IL-17B: interleukin 17B
IL-17R: IL-17 receptor
rIL-17B: recombinant human IL-17B
OS: overall survival
RT-qPCR: real-time quantitative PCR
TN: triple negative
