# Supplementary material for: The IL-17B-IL-17 receptor B pathway promotes resistance to paclitaxel in breast tumors through activation of the ERK1/2 pathway
Source: Oncotarget. 2017 Dec 6;8(69):113360–72. doi: 10.18632/oncotarget.23008 (PMC5768333; doi:10.18632/oncotarget.23008)
Supplement: Supplementary file 1 [file oncotarget-08-113360-s001.pdf]

The IL-17B-IL-17 receptor B pathway promotes resistance to paclitaxel in breast tumors through activation of the ERK1/2 pathway

SUPPLEMENTARY MATERIALS

Supplementary Table 1: Characteristics of breast cancer patients' cohort

	IL17B sample number	IL17RB sample number
Background	24	24
Tumor	86	104
**NPI Stage**		
NPI-1	42	51
NPI-2	30	35
NPI-3	10	14
**Grade**		
Grade 1	11	19
Grade 2	24	34
Grade 3	49	49
**TNM stage**		
TNM 1	40	54
TNM 2	31	35
TNM 3	6	6
TNM 4	3	4
TNM 2,3,4	40	45
**Prognosis**		
Disease free	61	72
With metastasis	6	7
With local recurrence	3	5
Died of breast cancer	10	13
All breast cancer related incidence	19	25
ER negative	55	63
ER positive	25	31
ER beta negative	62	75
ER beta positive	20	21

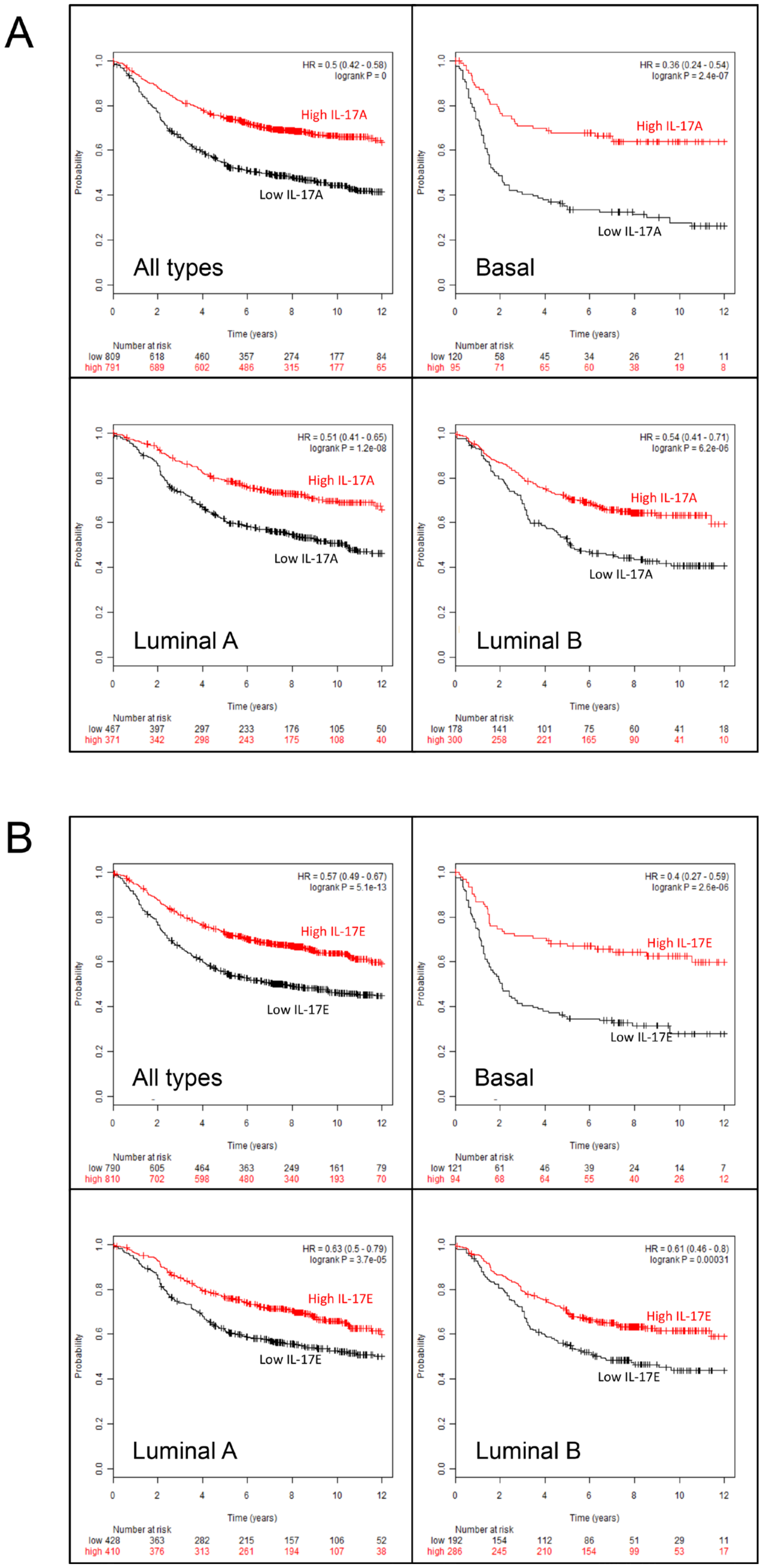

Supplementary Figure 1: Correlations between IL-17A or IL-17E expression and clinical outcome breast cancer patients. (**A, B**) Prognostic effect of IL-17A (A) and IL-17E (B) expression in the whole population and in patients with basal-like, luminal A or luminal B breast cancer assessed using the Kaplan-Meier method. Data were compared with the log-rank test. The median was taken as threshold for high IL-17A or IL-17E expression. Cohort size = 1809 patients, database 2012, collection of Affymetrix chips.

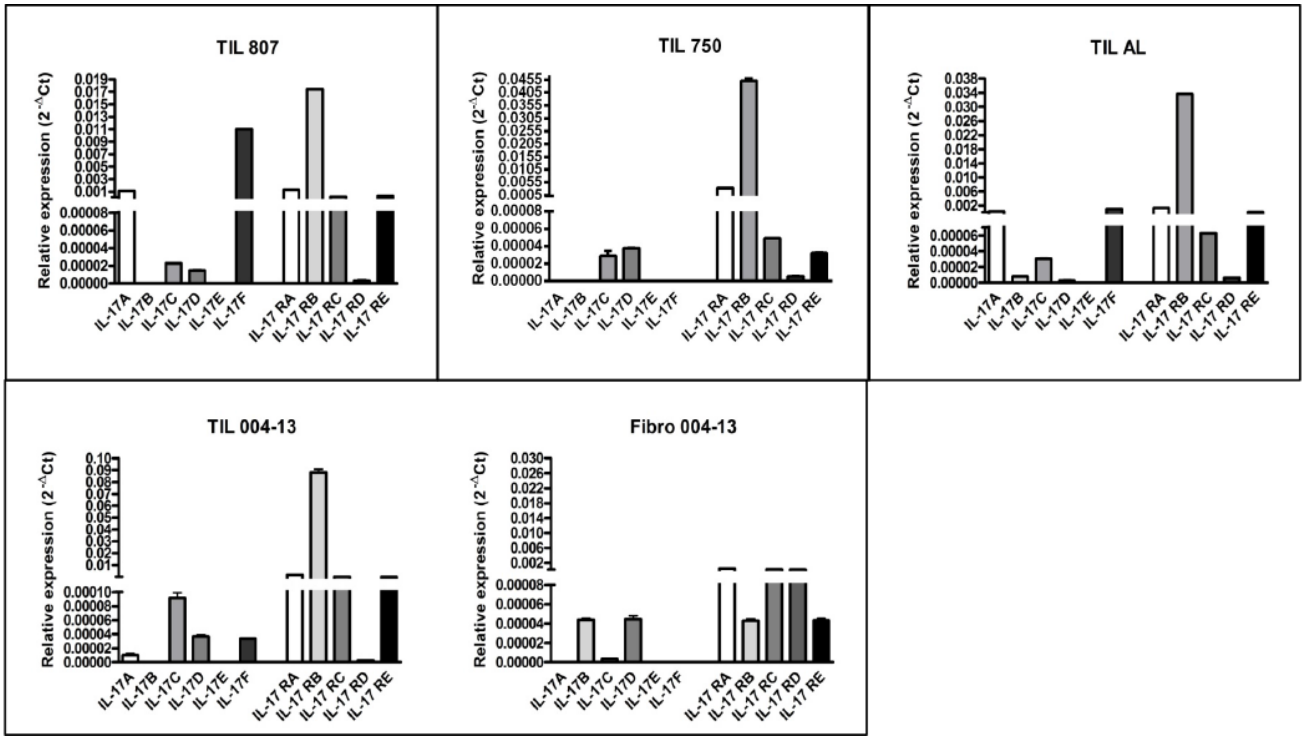

Supplementary Figure 2: Expression of IL-17 cytokines and their receptors in breast tumor infiltrated lymphocytes. Tumor infiltrating lymphocytes (TIL) and cancer-associated fibroblasts (fibro) were isolated from fresh biopsies and mRNA expression for IL-17 cytokines (IL-17A, IL-17B, IL-17C, IL-17D, IL-17E and IL-17F) and their receptors (IL-17RA, IL-17RB, IL-17RC, IL-17RD and IL-17RE) analyzed by qRT-PCR. Expression was normalized to the GAPDH mRNA expression. Data are the mean ± SEM of one or two experiments performed in duplicate.

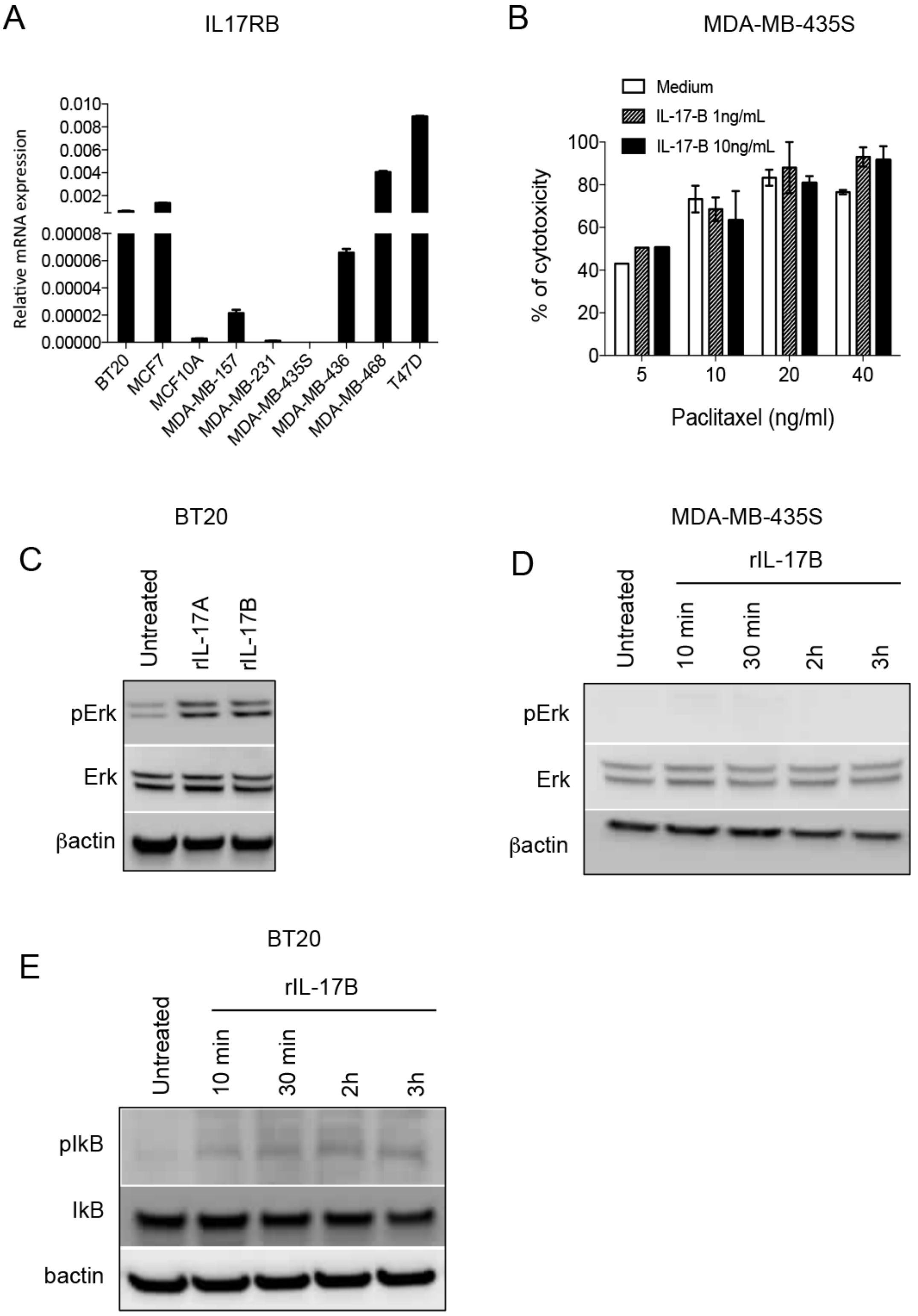

Supplementary Figure 3: Analysis of IL-17RB expression, IL-17B-induced chemoresistance and signaling pathways in breast cancer cell lines. (**A**) Comparison of IL-17RB expression in breast cancer cell lines determined by qRT-PCR. Expression was normalized to the GAPDH mRNA expression. (**B**) Percentages of drug-induced cell cytotoxicity in MDA-MB-435S cells cultured in FCS-free medium completed or not with 1 or 10 ng/mL rIL-17B for 24 h and treated with paclitaxel at concentrations ranging from 5 µg/mL to 40 µg/mL for 7 h. (**C, D**) Western blot analysis of ERK1/2 phosphorylation (pErk) in BT20 stimulated or not with 10 ng/mL rIL-17A or rIL-17B for 30 min (C) or in MDA-MB-435S (D) for different times. Total ERK and β-actin were used as protein loading control. (**E**) Western blot analysis of IκB phosphorylation (pIκB) in BT20 stimulated or not with 10 ng/mL rIL-17B for different times. Total IκB and β-actin were used as protein loading control. Results are representative of at least 2 experiments.

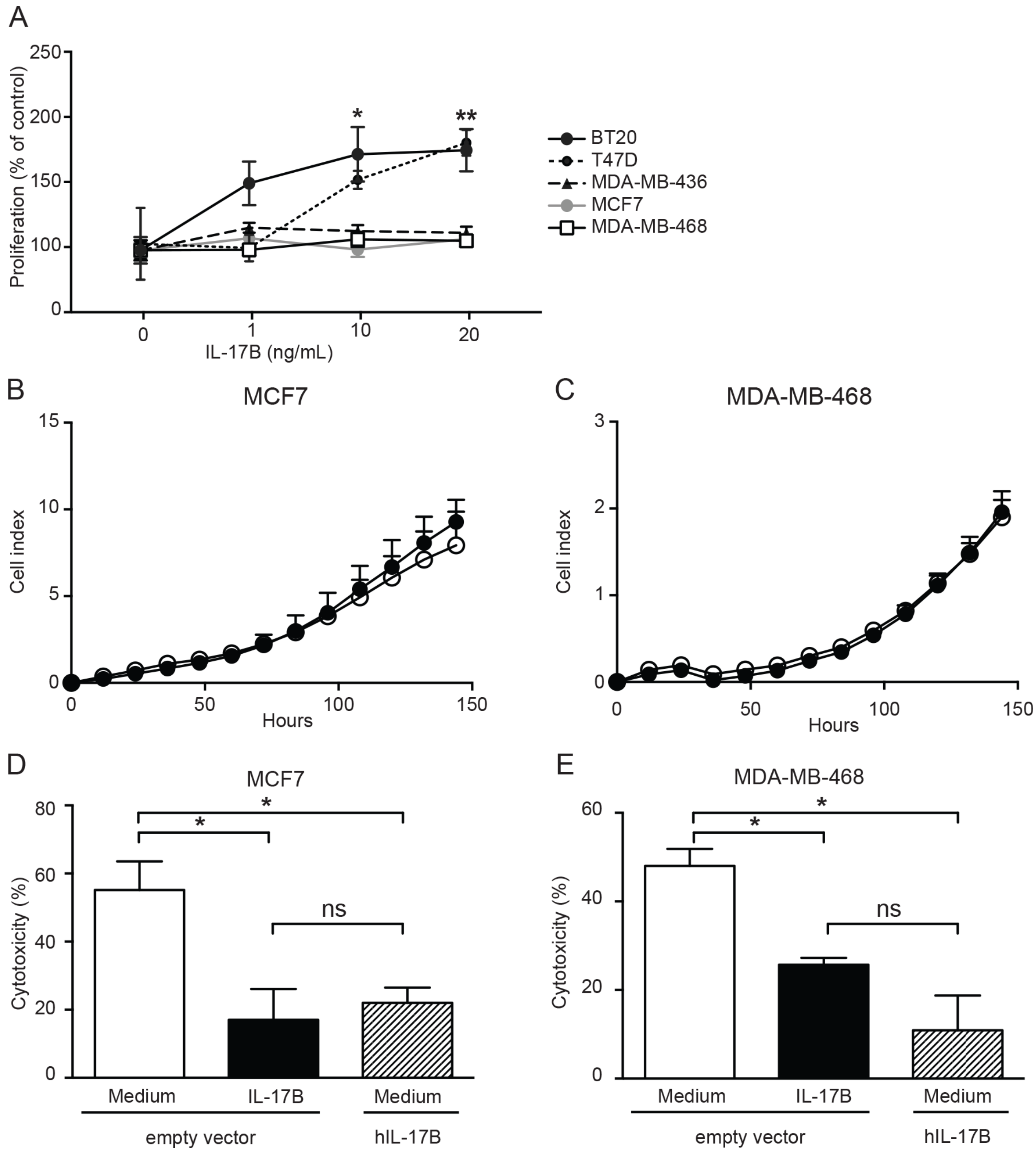

Supplementary Figure 4: IL-17B promotes resistance to paclitaxel independently of its effect on cell proliferation. (**A**) Cell proliferation determined by ^{3}H-TdR uptake in MDA-MB-468, BT20, MCF7, T47D and MDA-MB-436 breast cancer cells lines incubated with recombinant human IL-17B (0 to 20 ng/ml) for 72 h. Experiments were performed in hexaplicate ($n = 2$ for MDA-MB-468, BT20, and MDA-MB-436; $n = 1$ for T47D and MCF7). (**B**, **C**) Cell proliferation of MCF7 (B) and MDA-MB-468 (C) cells overexpressing (hIL-17B) or not (empty vector) human IL-17B monitored with the Xcelligence system every 3 h and up to 150 h. (**D**, **E**) Percentages of drug-induced cell cytotoxicity in MCF7 (D) cells and MDA-MB-468 (E) cells expressing hIL-17B incubated with 5 µg/mL or 40 µg/mL paclitaxel respectively for 7 h compared with parental cells and cells transfected with empty vector incubated or not with rIL-17B. For A, multi-comparison was done with the Kruskal-Wallis test with Dunn's post-test and for (D, E), the Student's *t*-test was used to compare control and treatment groups. $^*p < 0.05$.
